# Pruritogenic molecules in the skin of patients with dermatomyositis

**DOI:** 10.3389/fmed.2023.1168359

**Published:** 2023-05-11

**Authors:** Anett Vincze, Erika Herczeg-Lisztes, Katalin Szabó, Tibor Gábor Béldi, Melinda Nagy-Vincze, Ágnes Pór, József Varga, Katalin Dankó, Tamás Biró, Balázs István Tóth, Zoltán Griger

**Affiliations:** ^1^Division of Clinical Immunology, Department of Internal Medicine, Faculty of Medicine, University of Debrecen, Debrecen, Hungary; ^2^Laboratory for Cellular and Molecular Physiology, Department of Physiology, Faculty of Medicine, University of Debrecen, Debrecen, Hungary; ^3^Department of Pathology, Gyula Kenézy University Hospital, University of Debrecen, Debrecen, Hungary; ^4^Division of Nuclear Medicine, Department of Medical Imaging, University of Debrecen, Debrecen, Hungary; ^5^Department of Immunology, Faculty of Medicine, University of Debrecen, Debrecen, Hungary

**Keywords:** dermatomyositis, inflammatory myopathies, itch, pruritus, TRP channels, TNF-α, IL-6

## Abstract

**Introduction:**

Pruritus is a common excruciating symptom in systemic autoimmune diseases such as dermatomyositis (DM) but the pathogenesis is not fully understood. We intended to investigate the targeted expression analysis of candidate molecules involved in the development of pruritus in lesional vs. non-lesional skin samples of patients affected with active DM. We looked for correlations between the investigated pruriceptive signaling molecules, disease activity, and itching sensation of DM patients.

**Methods:**

Interleukins (IL-33 and IL-6), tumor necrosis factor α (TNF-α), peroxisome proliferator-activated receptor γ (PPAR-γ), and ion channels belonging to the transient receptor potential (TRP) family were analyzed. The expression of TNF-α, PPAR-γ, IL-33, IL-6, and TRP channels in lesional DM skin was evaluated by RT-qPCR and immunohistochemistry and was compared with non-lesional DM skin samples. Pruritus, disease activity, and damage of DM were evaluated by the 5-D itch scale and Cutaneous Dermatomyositis Disease Area and Severity Index (CDASI), respectively. Statistical analysis was performed with IBM SPSS 28 software.

**Results:**

A total of 17 active DM patients participated in the study. We could show that the itching score was positively correlated with the CDASI activity score (Kendall's tau-b = 0.571; *p* = 0.003). TNF-α gene expression was significantly higher in lesional DM skin than in non-lesional DM skin (*p* = 0.009) and differed in the subgroups of patients with different itch intensities (*p* = 0.038). The mRNA expression of lesional IL-6 correlated positively with 5-D itch and CDASI activity score (Kendall's tau-b = 0.585; *p* = 0.008 and 0.45; *p* = 0.013, respectively). TRPV4 expressions were positively correlated with CDASI damage score (Kendall's tau-b = 0.626; *p* < 0.001), but the mRNA expressions of the TRP family, PPAR-γ, IL-6, and IL-33 were not different in lesional and non-lesional samples. Immunohistochemistry analysis did not find significant alterations in the expressions of TNF-α, PPAR-γ, IL-6, and IL-33 in lesional and non-lesional regions.

**Discussion:**

Our results argue that cutaneous disease activity, TNF-α, and IL-6 might play a central role in DM-associated itch, while TRPV4 plays a central role in tissue regeneration.

## 1. Introduction

Dermatomyositis (DM) is a subtype of idiopathic inflammatory myopathies (IIMs) collectively known as myositis. It is a multisystem disorder caused by immune-mediated chronic inflammation with a wide variety of clinical manifestations including lung, joints, gastrointestinal tract, and cardiac abnormalities; however, its hallmark features are progressive symmetrical proximal muscle weakness and characteristic skin manifestations. Based on data from questionnaires, most of the patients suffer from pruritus (1), which has a significant impact on quality of life (QoL).

Pruritus, impairing the disease morbidity, is one of the most common symptoms in dermatological diseases, including systemic autoimmune diseases such as DM (2). It was found that DM produces more pruritus than cutaneous lupus erythematosus (3) and is worse than that reported for psoriasis, atopic dermatitis, Darier's disease, and vitiligo (2). The clinical management of itching conditions is one of the biggest challenges of daily dermatological practice even today (4). In the last decade, our knowledge about the molecular mechanisms of acute itch was greatly expanded (5–7); however, the development of chronic itch (>6 weeks) is not fully understood. In the skin, products of keratinocytes, mast cells, several immune cells, and additional metabolic and tissue factors can contribute to the excitation of pruriceptive sensory nerve endings. Moreover, sensory terminals can also release pro- and anti-pruritic factors (8). The key molecules playing potential roles in the pathogenesis of acute and chronic itch in inflammatory skin diseases are endothelin-1 (ET-1), interleukin 31 (IL-31), interleukin 6 (IL-6), interleukin 17 (IL-17), interleukin 33 (IL-33), tumor necrosis factor α (TNF-α), thymic stromal lymphopoietin (TSLP), and peroxisome proliferator activator γ (PPAR-γ) that can activate directly or indirectly pruritic nerve endings. TNF-α was shown to demonstrate a critical role in acute and chronic itch in mice and targeting this molecule may be beneficial for itch treatment (9). Furthermore, in psoriasis, the PPAR-γ agonists have been recently shown to diminish pruritus (10).

The ion channels involved in the initial depolarization of sensory neurons are considered amplifiers of pruriception. The best studied of these ion channels mostly belong to the transient receptor potential (TRP) family of ion channels and show significant overlap with those involved in nociception. This includes transient receptor potential vanilloid 1 (TRPV1), transient receptor potential ankyrin 1 (TRPA1), and other TRP members (8). Many of these abovementioned mediators of itch are related to TRP channels. However, in DM, the connection between the sensation of itch and the roles of the TRP ion channels, TNF-α, PPAR-γ, and other pruriceptors was poorly examined yet and data from primary human skin samples are scarce.

Therefore, our present study was a cross-sectional cohort study, in which we aimed at discovering the specific cellular signaling pathways in the development of DM-associated itch. We aimed at answering the following specific research questions: (1) Is there a connection between pruritus and cutaneous disease activity of dermatomyositis? (2) Can previously identified DM-associated cytokines and inflammatory mediators contribute to the development of itch? and (3) Which pruritogenic gene expression pattern is associated with pruritus and disease activity in lesional DM skin compared to non-lesional DM skin?

## 2. Patients and methods

This scientific cross-sectional study was conducted on our initiative, in 17 consecutive patients with DM under the care of the National Myositis Center, in the Division of Clinical Immunology, Faculty of Medicine, at the University of Debrecen, Hungary between February 2017 and September 2021. Written informed consent was obtained from all the subjects. This study is ethically compliant and was carried out in compliance with the Declaration of Helsinki. The eligibility criteria were the diagnosis of probable or definitive dermatomyositis based on Bohan and Peter (11) and/or 2017 EULAR/ACR classification criteria (12) with active skin symptoms. Cancer-associated dermatomyositis or patients with other conditions associated with itch (cholestatic liver diseases, infections, chronic renal failure, and other systemic skin diseases) were excluded. The global disease activity of DM was evaluated with the physician global visual analog scale (VAS) (13), and the cutaneous activity was determined by the Cutaneous Dermatomyositis Disease Area and Severity Index (CDASI), which is a validated disease severity score of DM (14). CDASI activity score ranges from 0 to 100, and the damage score ranges from 0 to 32. Muscle weakness was assessed with a manual muscle test (MMT) (15). The severity of pruritus was assessed with a 5-D itch scale (ranging from 5 to 25) (16). The 5-D itch scores were divided into three groups for analysis: no, or mild pruritus (score 5–10), moderate pruritus (score 11–15), and severe pruritus (score 16–25). Laboratory tests included the serum level of creatine kinase (CK), lactate dehydrogenase (LDH), aspartate transaminase (AST/GOT), glutamate–pyruvate transaminase (ALT/GPT), C-reactive protein (CRP), erythrocyte sedimentation rate (ESR), and creatinine.

### 2.1. Skin biopsy

From the 17 DM patients, a lesional (consisting of erythematous patches) and a non-lesional site 4–4 mm punch biopsies were performed. The active lesional samples were collected from the upper region of the back in 14 patients, from the upper arm in two patients, and from the lateral upper thigh in one patient. Non-lesional samples were taken from the same anatomical region without DM involvement. Then punches were cut into two sections, one for RT-qPCR (tissue homogenization, total RNA isolation, and quantitative real-time PCR) and the other one for immunohistochemistry.

### 2.2. RT-qPCR

Tissue homogenization was performed with 400 Hz, 60 s × 4 times; one zirconium bead/tube in 800 μl of TRIzol reagent (Life Technologies Corporation, Foster City, CA, USA) using BeadBug homogenizer (Benchmark Scientific, Sayreville, NJ, USA). Total RNA was isolated according to the manufacturer's instructions and digested with recombinant RNase-free DNase-1 (Life Technologies) according to the manufacturer's protocol. After isolation, 1 μg of total RNA was reverse transcribed into cDNA using the high-capacity cDNA kit (Life Technologies, USA) following the manufacturer's protocol. Quantitative real-time PCR was performed on a LightCycler 384 well sequence detection system (Roche) by using a 5' nuclease assay. PCR amplification of the genes of interest (TRPV1, TRPV2, TRPV3, TRPV4, TRPM2, TRPM3, TRPC1, TRPC6, TRPA1, TNF-α, PPAR-γ, IL-6, and IL-33) was performed using specific TaqMan primers and probes using the TaqMan Gene Expression Master Mix Protocol (Life Technologies, USA).

### 2.3. Immunohistochemistry

The immunohistochemical investigation of IL-6 (Novus Biologicals, Littleton, USA), IL-33 (Boster Biological Technology, CA, USA), TNF-α (Sigma-Aldrich, MO, USA), and PPAR-γ (LifeSpan BioSciences, WA, USA) was performed on formalin-fixed paraffin-embedded skin samples taken from 15 patients diagnosed with dermatomyositis. The immunoreaction was completed on lesional and non-lesional and healthy skin areas originating from the same patient. Serial 4 μm thick sections were cut from paraffin blocks, and hereon heat-induced antigen retrieval was performed on slides. IL-6 epitopes were retrieved in 1 mM EDTA buffer (pH 8) and placed in a microwave oven for 20 min on 700W. IL-6 epitopes were retrieved in 1 mM EDTA buffer (pH 8) and applied in a microwave oven for 20 min on 700W. IL-33, TNF-α, and PPAR-γ were retrieved in EnVision FLEX Target Retrieval Solution High pH (DAKO, Glostrup, Denmark) in a water bath for 30 min at 95°C. Endogenous peroxidase activity was blocked with 3% H_2_O_2_ for 10 min. After blocking, tissue sections were incubated at room temperature with primary antibodies diluted in antibody diluent solution (DAKO, Glostrup, Denmark): IL-6 (mouse, clone: OTI3G9, 1:100, 60 min), IL-33 (mouse, clone: 12B3C4 1:100, 60 min), TNF-α (mouse, clone: M1-C4, 1:300, 60 min), and PPAR-γ (mouse, clone: 8D1H8F4, 1:75, 60 min). Sections were then incubated with the EnVision FLEX Labeled polymer-HRP anti-rabbit and anti-mouse System (DAKO, Glostrup, Denmark) at room temperature for 30 min with 3,3'-diaminobenzidine (DAB) visualization techniques. Cell nuclei were counterstained with hematoxylin, and tissue sections were finally mounted in a permanent mounting medium (Histolab, Göteborg, Sweden). Normal skin sections served as positive controls for IL-6, IL-33, and PPAR-γ detection. The colon sample diagnosed as ulcerative colitis was used to validate TNF-α expression. Negative controls were obtained by omitting the primary antibody in all cases. The analysis of expressions was performed semi-quantitatively with ImageJ 1.48v software (NIH). Two independent blinded reviewers (AP and ZG) measured the mean staining intensity (including different cell types) of each sample and scored it from 0 to 3 (0: no staining, 1: mild intensity, 2: moderate intensity, and 3: strong intensity). Their differences were resolved by repeated analysis and by consensus. Statistical analysis and “dot plot” diagrams were performed. Every dot represents the staining intensity of one patient's skin sample.

### 2.4. Statistical analysis

Statistical analysis was performed using the SPSS 28 statistical software. The normality of distributions in the case of continuous variables was tested using the Shapiro–Wilk test. Data with normal distribution were presented as mean and standard deviation (SD) with minimum and maximum values, and data with non-normal distribution as the median and interquartile range (IQR). Categorical variables were described using frequency (case number) and percentage. Wilcoxon exact test was applied for gene expression changes. Kendall's tau was used with calculations based on concordant and discordant pairs. For the pairwise comparison, Hochberg's correction was applied. For comparing the immunohistochemistry staining intensity, we used an independent sample *t*-test. In different itching subgroups, Kruskal–Wallis exact and Jonckheere–Terpstra exact tests were applied. During statistical analysis, a *p*-value of < 0.05 was regarded as statistically significant.

## 3. Results

### 3.1. Demographic data and organ involvement

**Figure 1:**
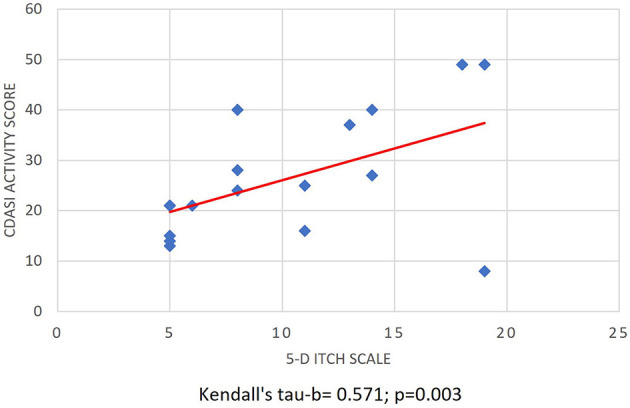
Association of cutaneous disease activity and itch intensity. Correlation of CDASI activity score and the 5-D itch score (Kendall's tau-b = 0.571; *p* = 0.003; *N* = 17) CDASI: Cutaneous Dermatomyositis Disease Area and Severity Index.

A total of 17 patients (six men and 11 women) participated in the study. Table 1 shows the demographic, basic clinical, laboratory parameters, and relevant organ involvements of the patients. The mean age at the biopsy was 58.82 ± 9.78 years (43–76), and the disease duration was 46 months (20–78). Every patient received the standard treatment for DM, including glucocorticoids and/or immunosuppressive agents. The global disease activity was mild-moderate in most patients (mean: 2.85 ± 0.99 (range 1–4), whereas the mean CDASI activity score was 26.24 ± 12.64 (8–49). Evaluating the pruritus sensation, the patient's median 5-D itching score was 8 (5–14). A total of 59% (10/17) of the patients experienced mild itch, 23.5% (4/17) moderate, and 17.65% (3/17) severe. The 5-D itch score correlated with CDASI activity (Kendall's tau-b = 0.571; *p* = 0.003; Figure 1), but not with CDASI damage score (*p* = 0.21). We could not detect a significant connection between the Global VAS and the 5-D itch score (Kendall's tau-*p* = 0.27).

**Table 1:** Clinical and laboratory parameters, organ manifestations, and relevant autoantibodies of the patients.

**Clinical parameters** ***N =*** **17**	
Age (years) ±SD (min-max)	58.82 ± 9.78 (43–76)
Male/Female (N)	6/11 (35.3–64.7%)
Disease duration (month) (IQR)	46 (20-78)
Pruritus (5D itch scale; min. 5.0, max. 20.0) (IQR)	8 (5-14)
CDASI-activity ±SD (min-max)	26.24 ± 12.64 (8-49)
CDASI-damage ±SD (min-max)	4.18 ± 2.53 (0–9)
MMT (Manual-Muscle-Test; max. 150) (IQR)	138 (133–140.5)
Physician Global Activity (Visual Analog Scale; max. 10.0) ±SD (min-max)	2.85 ± 0.99 (1-4)
**Laboratory parameters** ***N** =* **17**	
CK at biopsy (U/l) (IQR)	80 (51-105)
GOT - AST at biopsy (U/l) (IQR)	20 (17-25)
GPT - ALAT at biopsy (U/l) (IQR)	22 (17-39)
LDH at biopsy (U/l) (IQR)	264 (231-274)
Creatinine at biopsy (umol/l) (IQR)	62 (53-78)
CRP at biopsy (mg/l) (IQR)	3.98 (1.6-8.5)
ESR at biopsy (mm/h) ±SD (min-max)	21.19 ± 13.17 (2-50)
**Organ manifestations** ***N** =* **17**	
Skin involvement N (%)	17/17 (100%)
Muscle involvement N (%)	17/17 (100%)
Gastrointestinal involvement N (%)	8/17 (47.05%)
Cardiac involvement N (%):	2/17 (11.76%)
Pulmonary involvement N (%):	3/17 (17.64%)
Joint involvement N (%):	3/17 (17.64%)
**Autoantibodies** ***N** =* **17**	
Negative N (%)	7/17 (41.17%)
Anti-MDA5 N (%)	0/17 (0%)
Anti-TIF1γ N (%)	2/17 (11.76%)
Anti-NXP2 N (%)	1/17 (5.88%)
Anti-Mi2 N (%)	3/17 (17.64%)
Anti-SAE N (%)	2/17 (11.76%)
Anti-Jo-1 N (%)	1/17 (5.88%)
SSA/Ro52 N (%)	2/17 (11.76%)

SD, standard deviation, CDASI, Cutaneous Dermatomyositis Disease Area and Severity Index; CK, creatine kinase; GOT/AST, glutamic-oxaloacetic transaminase/aspartate aminotransferase; GPT/ALAT, glutamic pyruvic transaminase/alanine aminotransferase; LDH, lactate dehydrogenase; CRP, C-reactive protein; ESR, erythrocyte sedimentation rate; MMT, manual muscle test. Data are presented as mean and standard deviation (SD) with minimum and maximum values, or median and interquartile range (IQR). Categorical variables were described using frequency (case number) and percentage.

### 3.2. Targeted gene expressions of pruritogenic mediators

**Figure 2:**
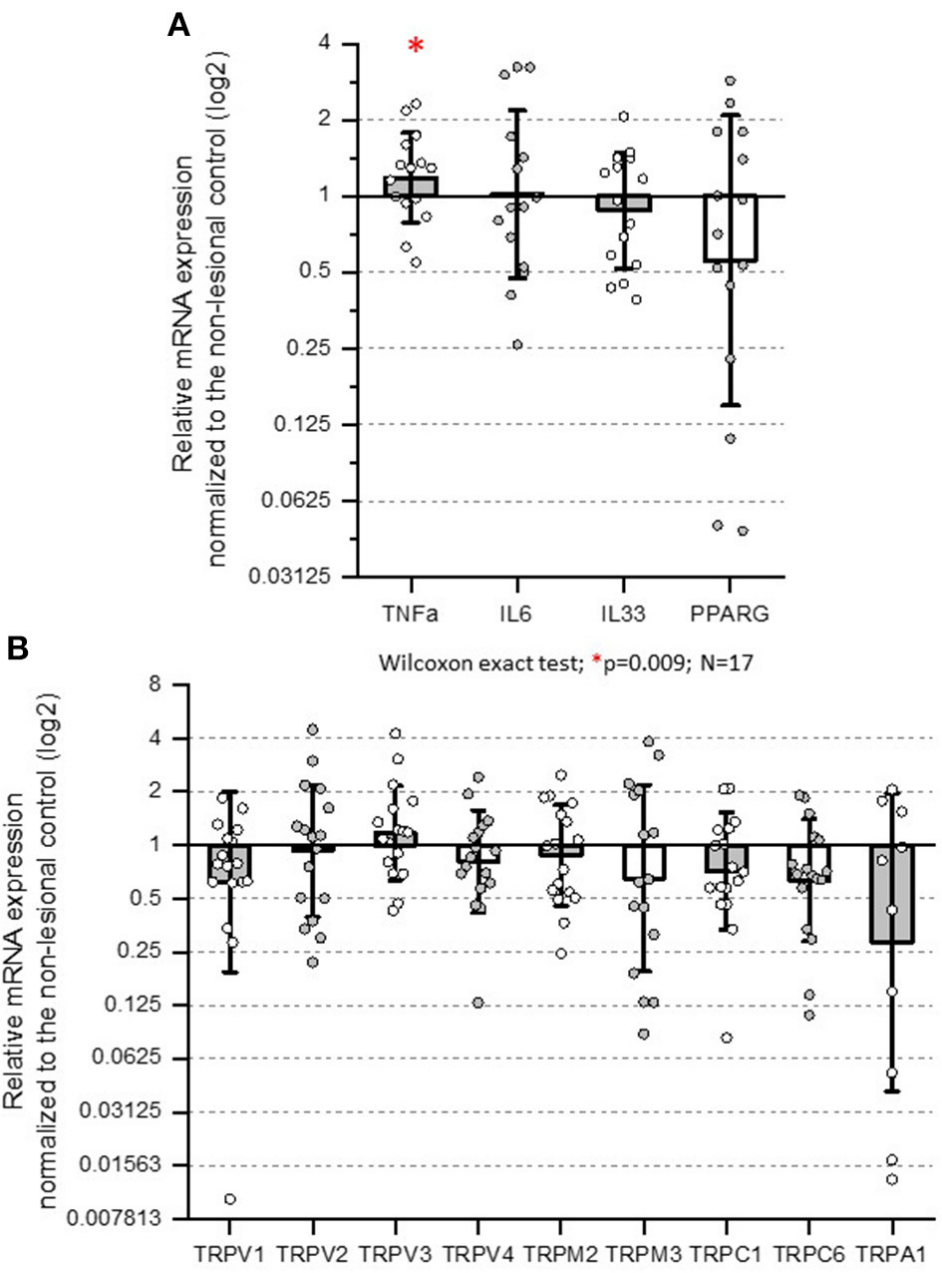
Normalized mRNA expressions of candidate genes participating in pruriception. Real-time PCR results of each gene of interest were normalized to the transcripts of GAPDH, and the values obtained from the lesional sites are shown as the fraction of the non-lesional site of the same patient (normalized mRNA expression). **(A)** The mRNA levels of TNF-α were significantly higher in lesional skin samples (Wilcoxon exact; *p* = 0.009). **(B)** The expression of the TRP ion channel family did not change significantly with Wilcoxon exact test. TRP: transient receptor potential.

**Figure 3:**
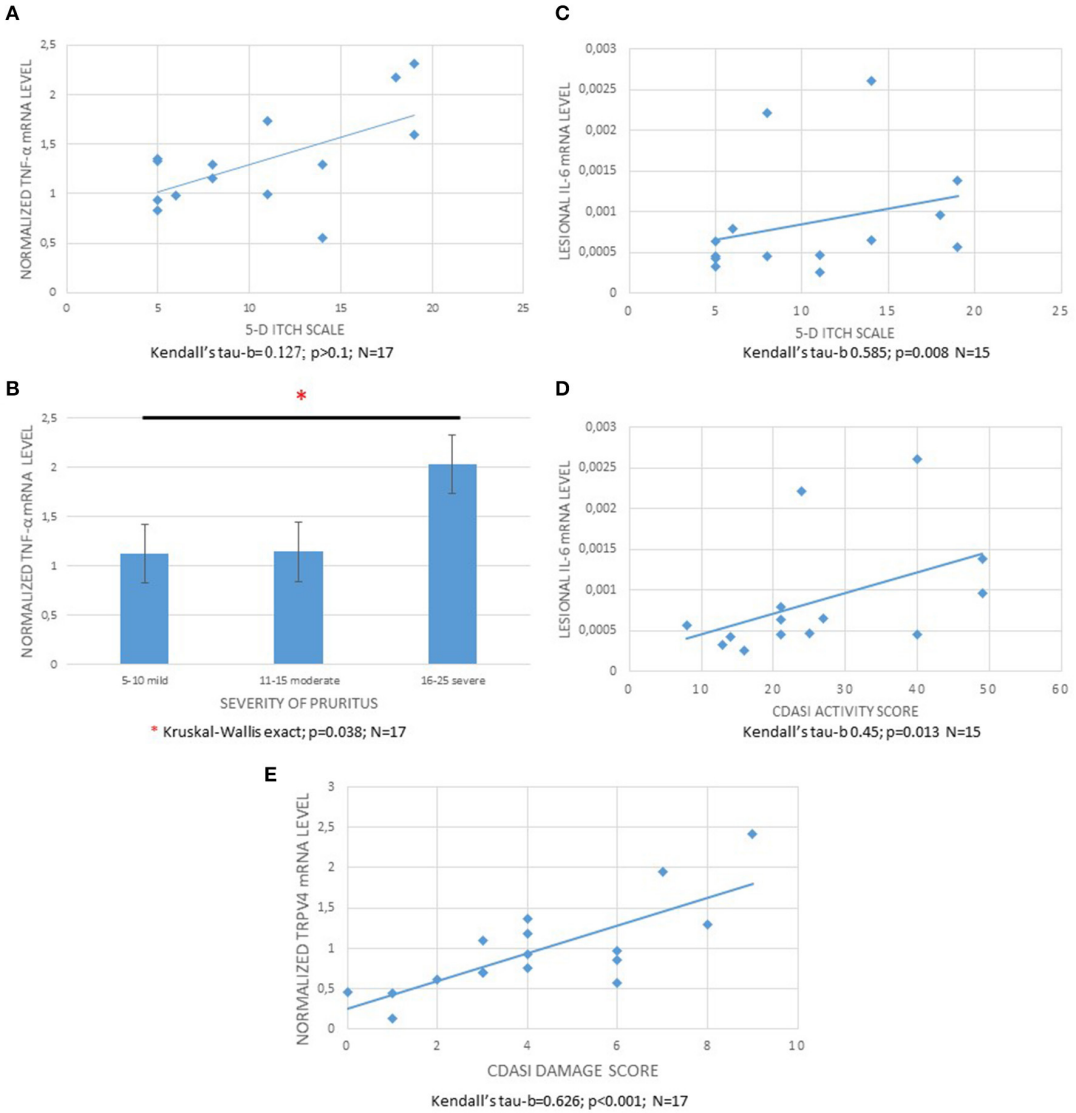
Association of normalized TNF-α, IL-6 levels with itch intensity and CDASI activity, and TRPV4 with CDASI damage score. **(A)** Correlation of 5-D itch scores and TNF-α mRNA levels normalized to the levels of non-lesional samples (Kendall's tau-b: 0.127; *p* > 0.1). **(B)** Normalized TNF-α mRNA levels in the skin samples of patients with different itch categories (mild: 5–10; moderate: 11–15; and severe: 16–25 on 5-D itch score, respectively) (Kruskal–Wallis exact; *p* = 0.038). **(C)** Correlation of 5-D itch scores and IL-6 mRNA levels in the lesional samples (Kendall's tau-b = 0.585; *p* = 0.008). **(D)**: Correlation of CDASI activity score and lesional IL-6 mRNA levels (Kendall's tau-b = 0.45; *p* = 0.013). **(E)** Correlation of normalized TRPV4 mRNA levels and CDASI Damage score (Kendall's tau-b = 0.626; *p* < 0.001). CDASI, Cutaneous Dermatomyositis Disease Area and Severity Index.

In 17 patients with active dermatomyositis, targeted gene expressions of pruritogenic mediators or receptors were evaluated in lesional and non-lesional skin samples (Figure 2). The mRNA levels of TNF-α were significantly higher in lesional skin samples than in non-lesional skin samples (Wilcoxon exact; *p* = 0.009). Furthermore, we could detect significantly different results of lesional TNF-α mRNA levels (normalized to levels of non-lesional samples) in the subgroups of patients with different itch intensities (Kruskal–Wallis exact; *p* = 0.038); however, the normalized TNF-α levels did not correlate with 5-D itch score (Kendall's tau-b = 0.127; *p* > 0.1; Figures 3A, B).

The mRNA levels of IL-6 in lesional DM skin samples were not significantly different in comparison with the non-lesional levels (Wilcoxon exact; *p* > 0.1; Figure 2A). However, lesional IL-6 mRNA levels correlated positively with 5-D itch and CDASI activity score (Kendall's tau-b = 0.585; *p* = 0.008 and 0.45; *p* = 0.013, respectively, Figures 3C, D). The normalized mRNA expressions of the PPAR-γ were numerically decreased in DM skin; however, this was not statistically significant (Wilcoxon exact; *p* > 0.1; Figure 2A). Furthermore, the normalized mRNA levels of PPAR-γ did not show a significant correlation with pruritus (Kendall's tau-b = −0.362; *p* = 0.058), and similarly, the levels were not different in the different itching intensity categories (Jonckheere–Terpstra exact; *p* = 0.053). Interestingly, the mRNA expressions of IL-33 were not significantly different between lesional and non-lesional skin samples.

The mRNA levels of TRP family members (TRPV1, TRPV2, TRPV3, TRPV4, TRPM2, TRPM3, TRPC1, TRPC6, and TRPA1) were not significantly different in the lesional and non-lesional samples (Figure 2B), and we could detect correlations neither between any of the TRP channels and the 5-D itch score nor between any TRP channel and the CDASI activity score. In contrast, we could detect a significant positive correlation between the normalized mRNA levels of TRPV4 and CDASI damage score (Hochberg's correction; Kendall's tau-b = 0.626; *p* < 0.001; Figure 3E).

### 3.3. Immunohistochemistry

**Figure 4:**
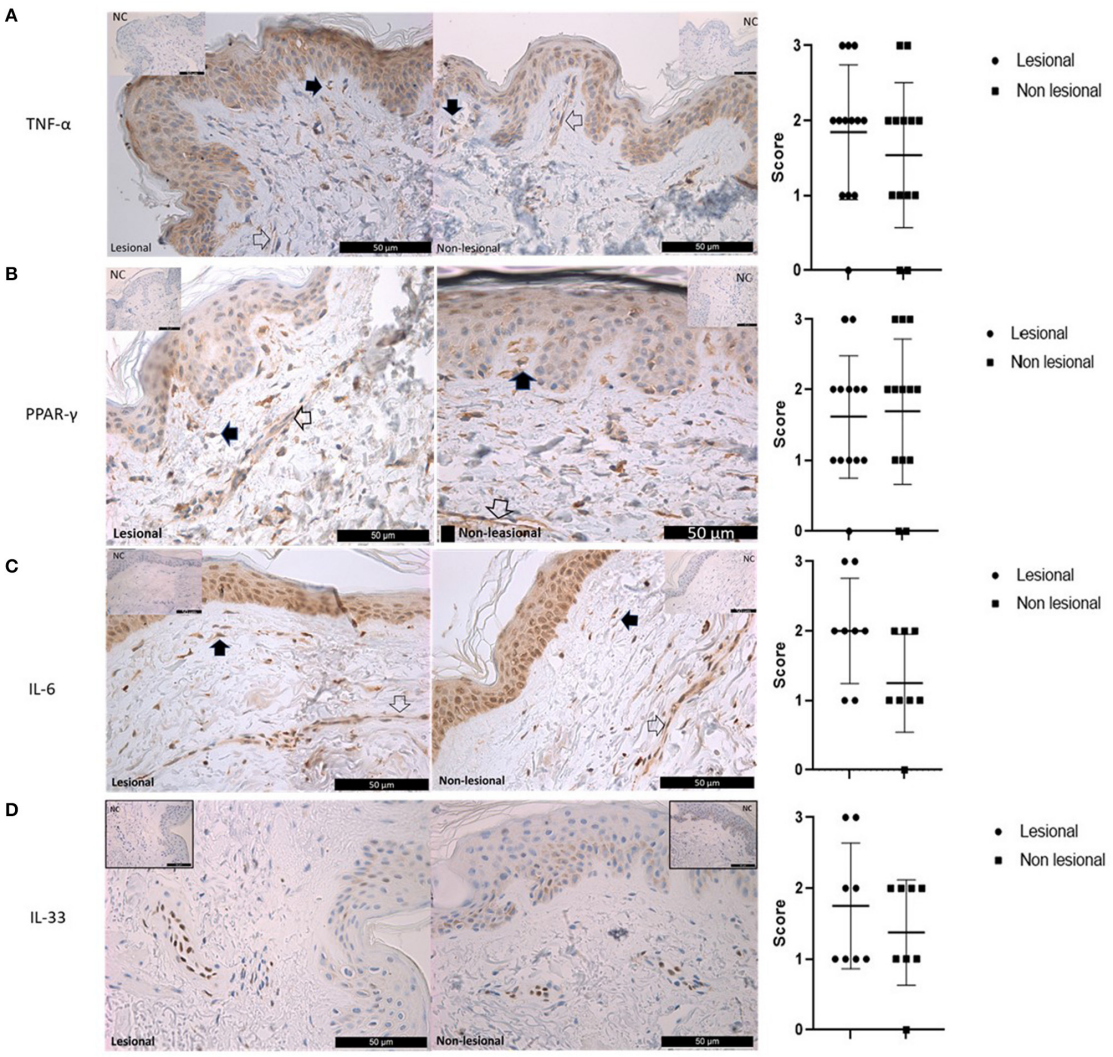
Immunohistochemistry analysis of TNF-α, PPAR-γ, IL-6, and IL-33 in lesional and non-lesional skin samples of patients with dermatomyositis. **(A)** TNF-α: Left panel: Expression of TNF-α in lesional and non-lesional dermatomyositis skin samples. The cytoplasmic expression is similar in the keratinocytes, dendritic cells (black arrow), and endothelial cells (empty arrow). Top right and left corners: Negative controls were obtained by omitting the primary antibody. Right panel: Statistical analysis of semi-quantitative measurements; (independent sample *t*-test; *p* > 0.1). **(B)** PPAR-γ: Left panel: Expression of PPAR-γ in lesional and non-lesional dermatomyositis skin samples. There is no difference between the lesional and non-lesional DM skin samples in the expression of the PPAR-γ. Empty arrow: endothelial cells. Black arrow: dendritic cells. Top right and left corners: Negative controls were obtained by omitting the primary antibody. Right panel: Statistical analysis of semi-quantitative measurements; (independent sample *t*-test; *p* > 0.1). **(C)** IL-6: Left panel: Expression of IL-6 in lesional and non-lesional dermatomyositis skin samples. Strong cytoplasmic/nuclear reaction with IL-6 in the epidermal endothelial (empty arrow) and dendritic cells (black arrow). Top right and left corners: Negative controls were obtained by omitting the primary antibody. Right panel: Statistical analysis of semi-quantitative measurements; (independent sample *t*-test; *p* > 0.1). **(D)** IL-33: Expression of IL-33 in lesional and non-lesional dermatomyositis skin samples. Positive reaction was detected in the nucleus of endothelial cells with IL-33, but there was no significant difference between lesional and non-lesional DM skin samples. Top right and left corners: Negative controls were obtained by omitting the primary antibody. Right panel: Statistical analysis of semi-quantitative measurements; (independent sample *t*-test; *p* > 0.1).

In view of gene expression data, immunohistochemistry analysis was performed staining for TNF-α, PPAR-γ, IL-6, and IL-33. Histological analysis revealed subepidermal and perivascular inflammatory infiltrates in lesional samples of DM skin. The protein expression of the TNF-α was localized mainly in the cytoplasm of keratinocytes, dendritic, and endothelial cells (Figure 4A); however, the semi-quantitative expressions of the staining were similar in the lesional and non-lesional slides (independent sample *t*-test, *p* > 0.1). PPAR-γ was detected with various expressions in the dendritic and endothelial cells, in the epidermis, but there was no significant difference between the lesional and non-lesional samples (Figure 4B). Regarding IL-6, we could detect strong cytoplasmic reaction in the epidermis, endothelial, and dendritic cells; furthermore, IL-6 was stained in the sweat glands and sebaceous glands; however, the staining expression was not different in the lesional and non-lesional areas (Figure 4C). We detected a positive reaction in the nucleus of endothelial cells with IL-33, but there was no significant difference between lesional and non-lesional DM skin (Figure 4D).

## 4. Discussion

In the present study, targeted expressions of mediators and receptors, playing a crucial role in itch sensation, were assessed in a Hungarian cohort of DM patients. We can summarize our recent study as follows: (1) we confirmed that itch sensation was significantly associated with the cutaneous disease activity of DM determined by CDASI; (2) the mRNA expressions and immunohistochemistry analysis of the majority of examined pruritogenic mediators and receptors were not different in the lesional and non-lesional skin samples; and (3) TNF-α and IL-6 might contribute to the development of itch in patients with DM.

Dermatomyositis is one of the most obvious pruritus-associated conditions among systemic autoimmune diseases, involving the skin. In a study involving 191 DM patients, 90.6% of the participants had at least mild itch, and more than 50% had moderate or severe itch based on a visual analog scale (1). In addition, in another cohort, 84.6% of DM patients experienced pruritus, and pruritus produced an effect on daily living in the majority of the patients, even though the muscle disease was not active at the time of the survey (17). Literature data about the pathomechanism of itch in DM are limited, especially investigations on primary human samples are scarce. It seems that cutaneous disease activity and consequent inflammation of the skin are associated with itch sensation, arguing for the direct relationship between disease pathogenesis and pruritus. Our results demonstrated that the itch sensation of the patients was associated with the affected area and severity of cutaneous manifestations of DM (CDASI score) and not with the global disease activity or with CDASI damage score. These results are in line with previous literature reports (1) and suggest that itch associates dominantly with cutaneous disease activity and not with global disease activity (including muscle, pulmonary, cardiac, and other scores) or cutaneous damage. Moreover, it seems that skin-dominant DM (even without muscle involvement or activity) is a unique form within the IIM-s, and CDASI is an important tool to assess cutaneous disease activity besides International Myositis Assessment and Clinical Studies Group (IMACS) Core set measurements (including skin assessment within Extramuscular Assessment - Myositis Disease Activity Assessment Tool).

Tumor necrosis factor α is a key molecule in the pathogenesis of many systemic inflammatory diseases, and a wide range of well-known molecules are on the market for treatment, including TNF inhibitors. Our results, regarding the elevated TNF-α mRNA levels in affected DM skin, are arguing for an important role of the molecule in the pathogenesis of the diseases and consequently in pruritus. Furthermore, the levels of TNF-α were different in skin samples of patients with different itches; however, a direct association between normalized TNF-α levels and itching score was not detected. Elevated levels of expressions of TNF-α have been found in the serum and muscle fibers of patients with DM (18, 19), which led to some pilot studies with TNF inhibitors for the treatment of DM. However, the effectiveness of these agents is unclear. In some studies, TNF-α inhibitors produced contradictory results and, in some cases, even worsened cutaneous disease (20–22). Furthermore, there are case reports demonstrating DM development under anti-TNF treatment for pre-existing conditions (23–25). In contrast, in a large cohort, 60 refractory juvenile DM patients had received at least 3 months of adalimumab/infliximab treatment and compared to baseline; there were improvements at 6 and 12 months in skin disease, including calcinosis (26). The abovementioned literature data and our results might indicate that there could be some unknown factors determining disease subtypes with different pathomechanisms and thus the treatment effectivity of TNF inhibitors in skin-dominant DM patients. There are discrepancies between normalized mRNA levels and protein expressions of TNFα in the skin samples. These might indicate a post-transcriptional regulation of TNFα in dermatomyositis, alterations in protein degradation by the ubiquitin system, or other unknown mechanisms.

IL-6 is a proinflammatory cytokine, whose expression in the serum of DM patients was significantly higher than that of normal controls (27). We could not detect elevated mRNA levels of IL-6 in lesional samples; however, normalized IL-6 mRNA levels were associated with 5-D itch and CDASI activity score, which argue for a significant role of IL-6 in inflammation and in DM-related itch sensation. Furthermore, the serum levels of IL-6 were significantly correlated with the disease activity of DM patients (28), indicating an important role of IL-6 in disease pathogenesis. The elevated levels of IL-6 were detected in patients with severe uremic pruritus in comparison with uremic patients without pruritus (29). Interestingly, in a recently published phase 2 trial, an IL-6 inhibitor tocilizumab was safe and well-tolerated but did not meet the primary or secondary efficacy outcomes in refractory DM and PM patients; however, only eight DM patients received tocilizumab, and the effect of the drug on pruritus was not presented (30).

Our results demonstrated only tendencies toward negative associations between itching sensation and lesional mRNA levels; however, statistical significance was not found (*p* = 0.053 and 0.058, respectively). There is evidence indicating the involvement of PPAR-γ receptors in the pathogenesis of skin diseases (31–33). The antidiabetic agents, glitazones, are agonists of the nuclear receptor PPAR-γ, which have anti-inflammatory and immunomodulatory properties. They are attributed to the downregulation of inflammatory interleukins (IL-1β, IL-2, and IL-6) and TNF-α (34, 35). The advantageous property of PPAR-γ agonists on itching in various dermatological disorders has been reported by many clinical studies (36–38). PPAR-γ agonists, which are used in the treatment of diabetes mellitus, have been recently shown to diminish pruritus not only in animal models but also in patients suffering from psoriasis (2, 39). We believe that PPAR-γ might play a negative regulator role in DM-related pruritus, but further experiments with higher patient numbers are required to determine this function in itching pathogenesis.

One of the key molecules, which could participate in DM-associated itch, is IL-31. It was found to be upregulated in the DM skin and correlated with pruritus (1). Furthermore, small fiber neuropathies are thought to be a major cause of neuropathic itch (40). However, to our knowledge, there was no data in the literature about the role of the TRP family in DM-related pruritus. Based on our data, it seems that there is no direct connection between the itch sensation or cutaneous disease activity and the amount of different TRP channels in the affected skin since the mRNA levels were associated neither with any marker of disease activity nor with a 5-D itch score. In contrast, we could detect that the normalized expression of TRPV4 mRNA was associated with the CDASI damage score which might indicate that this channel participates in regenerating processes following cutaneous inflammation. This hypothesis is strengthened by the fact that TRPV4 activation accelerates barrier recovery, and the formation of intercellular junctions between keratinocytes since this channel is co-localized to adherent junction proteins such as E-cadherin and b-catenin (41).

The limitations of this study should be acknowledged. This study was a single-center study from a national myositis unit in Hungary; the number of participants was relatively low. The lower number of patients with mRNA examinations could be a cause for selection bias, and the determination of protein expression was performed using a semi-quantitative method. Furthermore, the patients involved in the study had background immunosuppressive medication, which might affect the results.

It can be concluded that itching sensation is a common and probably underestimated condition of DM patients, which is associated with cutaneous disease activity. It seems that the members of the TRP family are not contributing directly to DM-associated itch, but TRPV4 might participate in skin regeneration processes. Our experiments argue for a determining role of TNF-α and IL-6 in DM-associated pruritus, and further experiments are required to study the role of PPAR-γ. We believe that our findings may help the development of new treatments for DM-associated itch but further investigations are necessary to control this excruciating symptom.

## Data availability statement

The raw data supporting the conclusions of this article will be made available by the authors, without undue reservation.

## Ethics statement

The studies involving human participants were reviewed and approved by the Institutional Review Board of University of Debrecen (Ethical permission number: DE RKEB/IKEB 4860-2016 and OTH IF-1647-9/2016). The patients/participants provided their written informed consent to participate in this study.

## Author contributions

ZG: conceptualized the study and design. AV, EH-L, KS, KD, MN-V, ÁP, and ZG: contributed to data acquisition. AV and MN-V: performed the skin biopsies. AV and BT: prepared the figures and tables. EH-L, BT, and TB: contributed to gene expression analysis. ÁP and ZG: contributed to immunohistochemistry investigation, data interpretation, and drafted the manuscript. JV: contributed to statistical analysis. KS, MN-V, TB, BT, TB, and JV: revised the manuscript critically for important intellectual content. ZG: supervised the manuscript. All authors gave their final approval of the version of the manuscript to be published.
